# Restorative effects of alpha-1A adrenergic are detectable using T2* and targeted nanoparticles in a mouse myocardial infarction (MI) model

**DOI:** 10.1186/1532-429X-15-S1-P177

**Published:** 2013-01-30

**Authors:** Justin Lam, Yongquan Gong, Robert C Robbins, Paul C Simpson, Phillip C Yang, Rajesh Dash

**Affiliations:** 1 Medicine, Stanford University, Stanford, CA, USA; 2 Cardiac Surgery, Stanford University, Stanford, CA, USA; 3 Medicine and Cardiology, University of California, San Francisco, San Francisco, CA, USA

## Background

Apoptosis is believed to play a major role in the progressive weakening of the peri-infarct and remote zone myocardium after myocardial infarction (MI). Our laboratory previously developed an in vivo, MRI-detectable apoptosis probe. Annexin-V (ANX), which binds to cells undergoing apoptosis, was conjugated to superparamagnetic iron oxide (SPIO) nanoparticles, allowing for the non-invasive detection of early apoptotic cell populations (ANX-SPIO r1: 8.6 ± 0.61 mM-1 s-1 and r2: 326 ± 16 mM-1 s-1). In recent work, we demonstrated A61603 (A6), an α1-adrenergic receptor agonist, can rescue cardiac cells from apoptosis through activation of the cardio-protective ERK pathway. We tested whether pre-treatment with A6 was able to prevent the functional decline after MI via an anti-apoptotic mechanism, and whether T2* cardiac MRI, using ANX-SPIO, was sensitive to this dynamic protective effect. Our hypothesis was that A6 pre-treatment protects against MI-induced cardiomyopathy more effectively than A6 treatment after MI.

## Methods

Female fvb/n mice were divided into three experimental groups. The A6 PREMI group underwent subcutaneous pump implant that delivered A6 two days before LAD ligation. The A6 MI and vehicle (VEH) groups underwent MI surgery simultaneously with subcutaneous A6 or vehicle pump implantation. Pump rate was 10 ng/kg/day over a course of two weeks, and all groups had their pumps re-implanted after two weeks. Cardiac MRI (CMR) was performed at 1, 2, and 4 weeks post MI. ANX-SPIO was delivered via tail vein one day prior to CMR to simultaneously assess left ventricular function and apoptosis (using T2* signal loss as a marker of apoptotic activity) in vivo. Delayed gadolinium enhancement MRI (DEMRI) and T2* were assessed by GE 3T Signa Excite HD MRI: GRE TR 100ms, TE 5-20ms, FA60, 256x256, FOV 4, ST 0.8mm, NEX 6. Myocardial caspase 3/7 activities were also assessed in each group.

## Results

**Figure 1:**
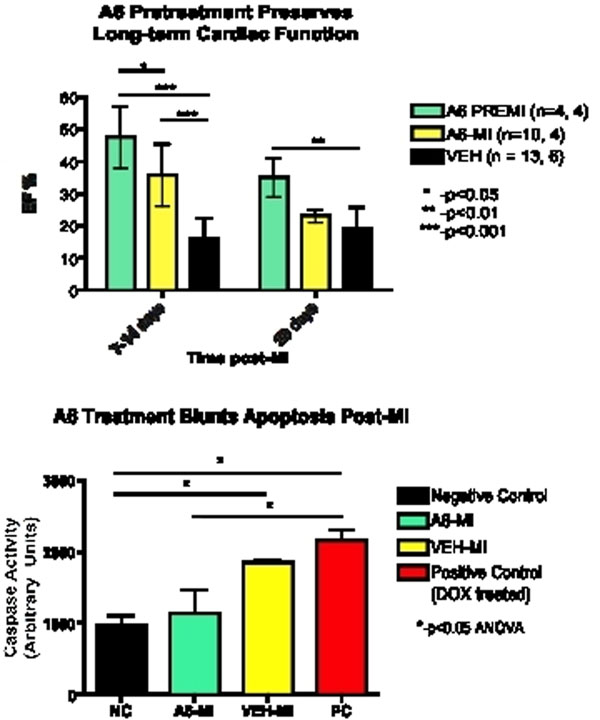
A) Cardiac ejection fraction (EF) at early (1-2 weeks) and late (4 week) timepoints post-MI. A6 PREMI exhibits significantly greater preservation of EF compared to A6-MI and VEH treated mice at an early timepoint. At late followup (4 weeks), only A6 PREMI animals exhibited significantly higher EF than VEH-treated controls, indicating the importance of pre-treatment with A6. B) Caspase activity blunted by A6 treatment. Caspase activity measured in hearts after two weeks of continuous treatment with either A6 (10ng/kg/d) or VEH solution. Note the significantly reduced caspase activity in A6-MI hearts compared to positive control (PC, Doxorubicin-treated hearts), whereas VEH-MI hearts were not significantly different from PC hearts. In addition, negative control hearts had significantly lower caspase activity levels than VEH-MI hearts, but not A6-MI hearts.

**Figure 2:**
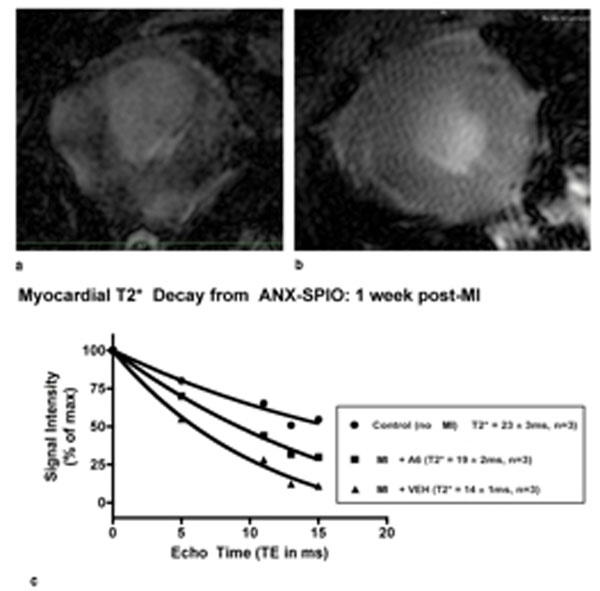
a) CMR images of short-axis VEH-MI heart showing T2* signal loss from ANX-SPIO update in the remote and border zones (arrows). b) CMR images of A6-MI heart showing visibly ANX-SPIO T2* signal loss. c) T2* decay curves showing more rapid T2* decay in VEH-MI hearts compared to A6-MI hearts.

At 1-2 weeks post-MI, A6 PREMI EFs (48±10%, n=4) were significantly higher than A6 MI EFs (36±10%, n=10), and both were significantly higher than VEH (16±6%, n=13) mice (p<0.001). At 4 weeks, however, only A6PREMI mice (35±6%) exhibited sustained cardiac function, with a significantly higher ejection fraction than VEH mice (19±6) at week 4 (p<0.01) (Figure 1A). The preserved EF in the A6-MI mice was associated with significantly lower myocardial Caspase 3/7 activity (Figure 1B). Upon T2* decay assessment, A6-treated mice showed significantly (p<0.05) less T2* signal loss after ANX-SPIO delivery compared to VEH-treated mice at 1 week post MI (A6 T2*: 19±2ms; VEH T2*: 14±1, n=3), reflecting less myocardial uptake of ANX-SPIO and therefore less cardiac cell apoptosis in A6-treated hearts (Figure 2).

## Conclusions

A6 pretreatment attenuates the decline in cardiac function post-MI, compared to A6 post-treatment. T2* Cardiac MRI using ANX-SPIO is able to detect the anti-apoptotic benefits of A6 treatment in vivo.

## Funding

NIH/NHLBI (PY, RD).

